# Atypical bronchial carcinoid with postobstructive mycobacterial infection: case report and review of literature

**DOI:** 10.1186/s12890-019-0806-x

**Published:** 2019-02-15

**Authors:** Abdulrahman Hakami, Evita Zwartkruis, Teodora Radonic, Johannes M. A. Daniels

**Affiliations:** 1 Department of Pulmonary Medicine, Amsterdam University Medical Center, Amsterdam, The Netherlands; 2 Department of Pathology, Amsterdam University Medical Center, Amsterdam, The Netherlands

**Keywords:** Carcinoid, Neuro-endocrine tumor, *Mycobacterium tuberculosis*, Nontuberculous mycobacteria, Postobstructive pneumonia

## Abstract

**Background:**

Pulmonary carcinoids are included in the group of neuroendocrine tumors (NET) and derive from pulmonary neuroendocrine cells. The incidence of these tumors is increasing, but disease awareness remains low among clinicians. The synchronous presentation of lung cancer and mycobacterial infection is well known but the combination of pulmonary carcinoid and mycobacterial infection is rare.

**Case presentation:**

We treated a 45-year-old female who presented with recurrent pneumonia. Chest X-ray showed a consolidation in the left upper lobe. The patient was treated with various courses of antibiotics without full recovery after six months. Computed tomography (CT) scan demonstrated a central mass in the left upper lobe. Bronchoscopy revealed an endobronchial, well-defined lesion that totally obstructed the left upper lobe bronchus. Bronchial biopsy showed typical carcinoid tumor. Rigid bronchoscopy with electrocautery was attempted, but we were unable to radically remove the tumor. Therefore lobectomy was performed. The surgical pathology specimen showed atypical bronchial carcinoid and consolidations in the lung parenchyma with granulomatous inflammation distally of the bronchial obstruction. Ziehl-Neelsen staining demonstrated acid fast bacilli indicative of mycobacterial infection.

**Conclusions:**

This case history illustrates the importance of careful surgical pathologic examination, not only of the resected tumor, but also of the postobstructive lung parenchyma. Specific postobstructive infections such as tuberculosis or nontuberculous mycobacteria (NTM) can have clinical implications.

## Introduction

Pulmonary carcinoids are NET arising from Kultchitzsky cells and 25% occur in the respiratory tract [1]. Pulmonary carcinoids represent about 1–2% of all primary lung tumors with an age-adjusted incidence rate ranging from 0.2 to 2/100000 population/year in both US and European countries [2].

Carcinoid tumors are divided into low-grade (typical) and intermediate-grade (atypical), based on mitotic activity and presence of necrosis. The location of pulmonary carcinoids can be central or peripheral, although the majority is located centrally [2, 3]. Respiratory symptoms are generally present only in central lesions, while peripheral forms are generally discovered as an incidental finding. The most frequent respiratory symptoms are recurrent chest infections, cough, hemoptysis, chest pain, dyspnea, and wheezing.

The cause of pulmonary carcinoid is unknown and there is no strong association with smoking or environmental carcinogens [1, 2].

## Case report

**Fig. 1:**
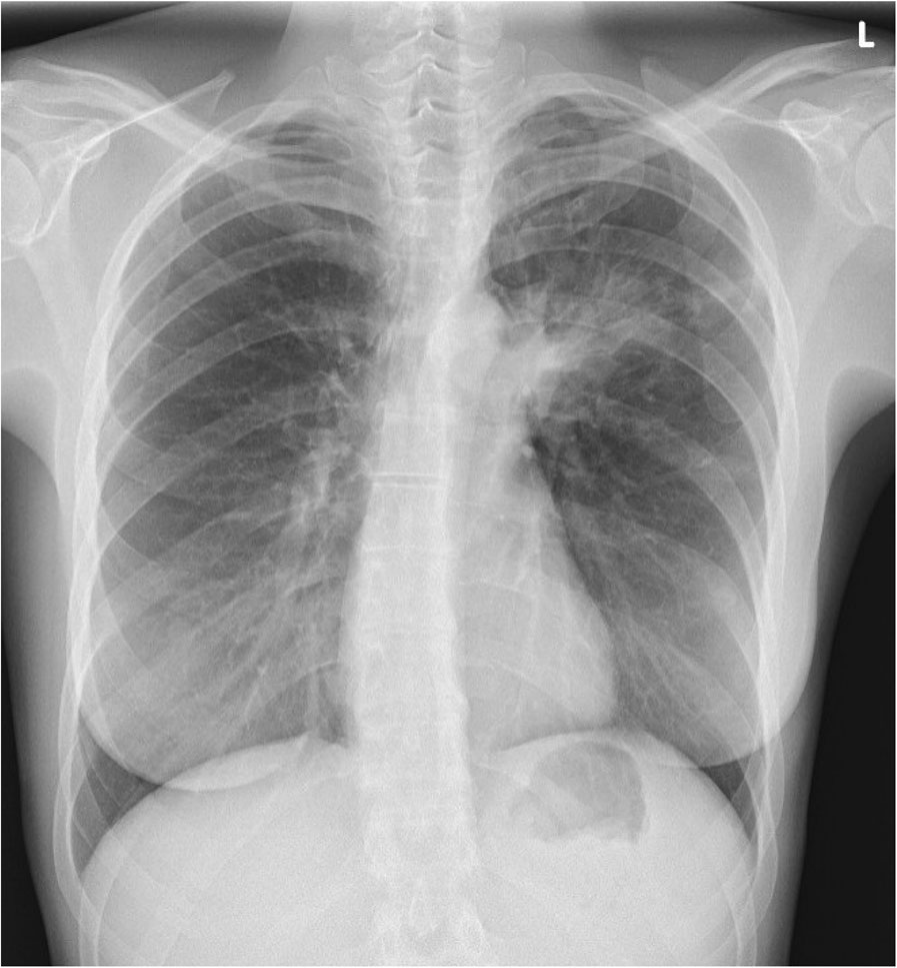
Chest X-ray showing a consolidation in the left hilum and left upper lobe

**Fig. 2:**
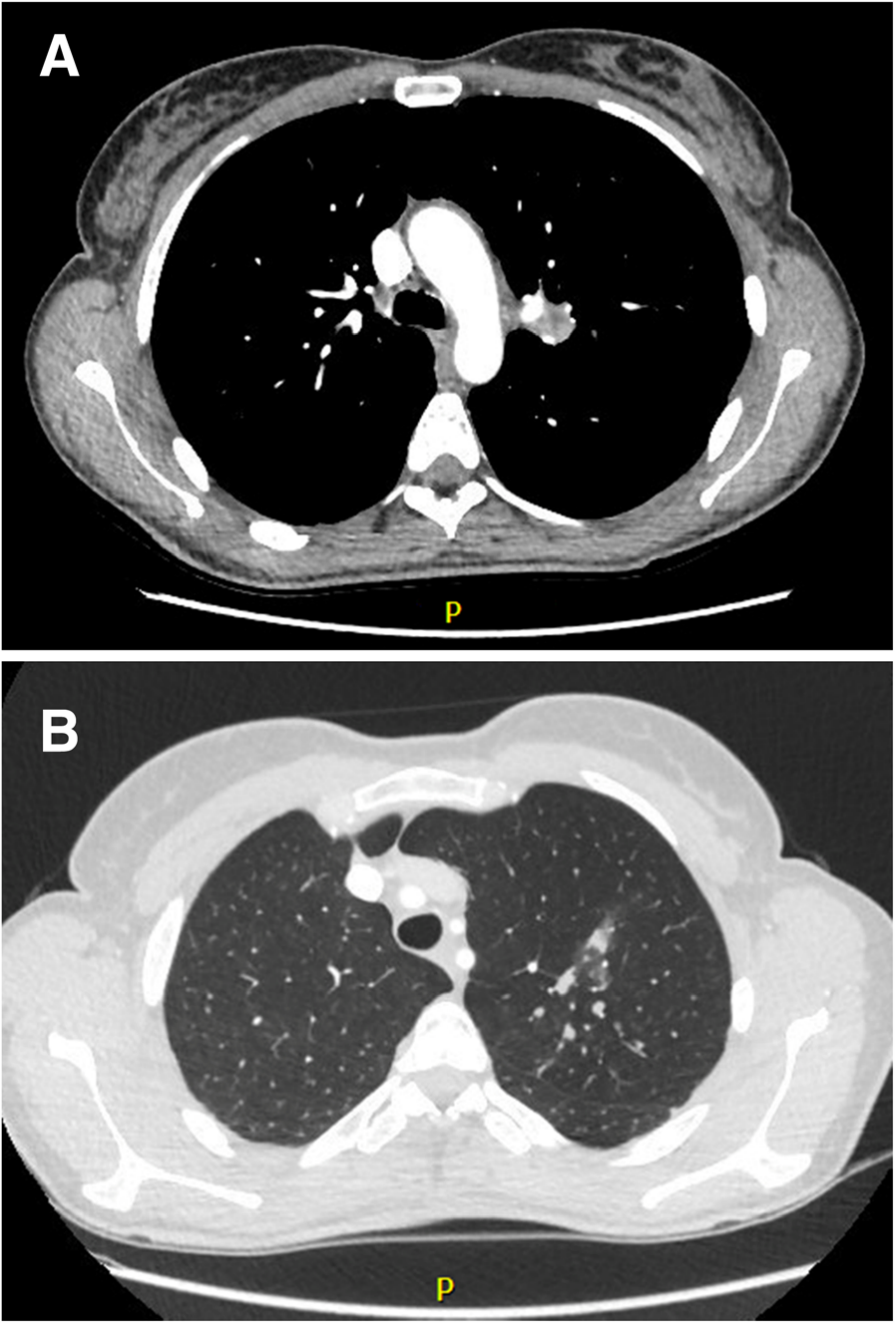
Computed tomography (CT) of the thorax showing the bronchial lesion in the left upper lobe (**a**) and a tumor mass at the location of the left upper lobe bronchus and some consolidation in the lung parenchyma of the left upper lobe (**b**)

**Fig. 3:**
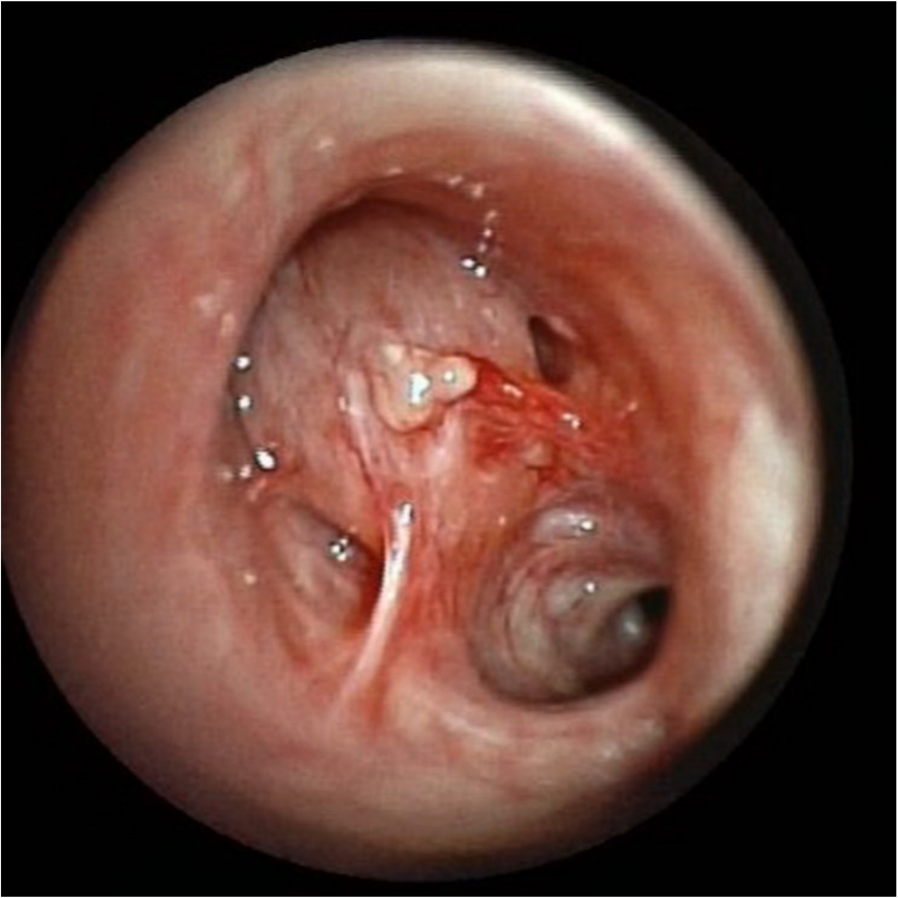
Rigid bronchoscopy demonstrates an endobronchial tumor in the left upper lobe bronchus

**Fig. 4:**
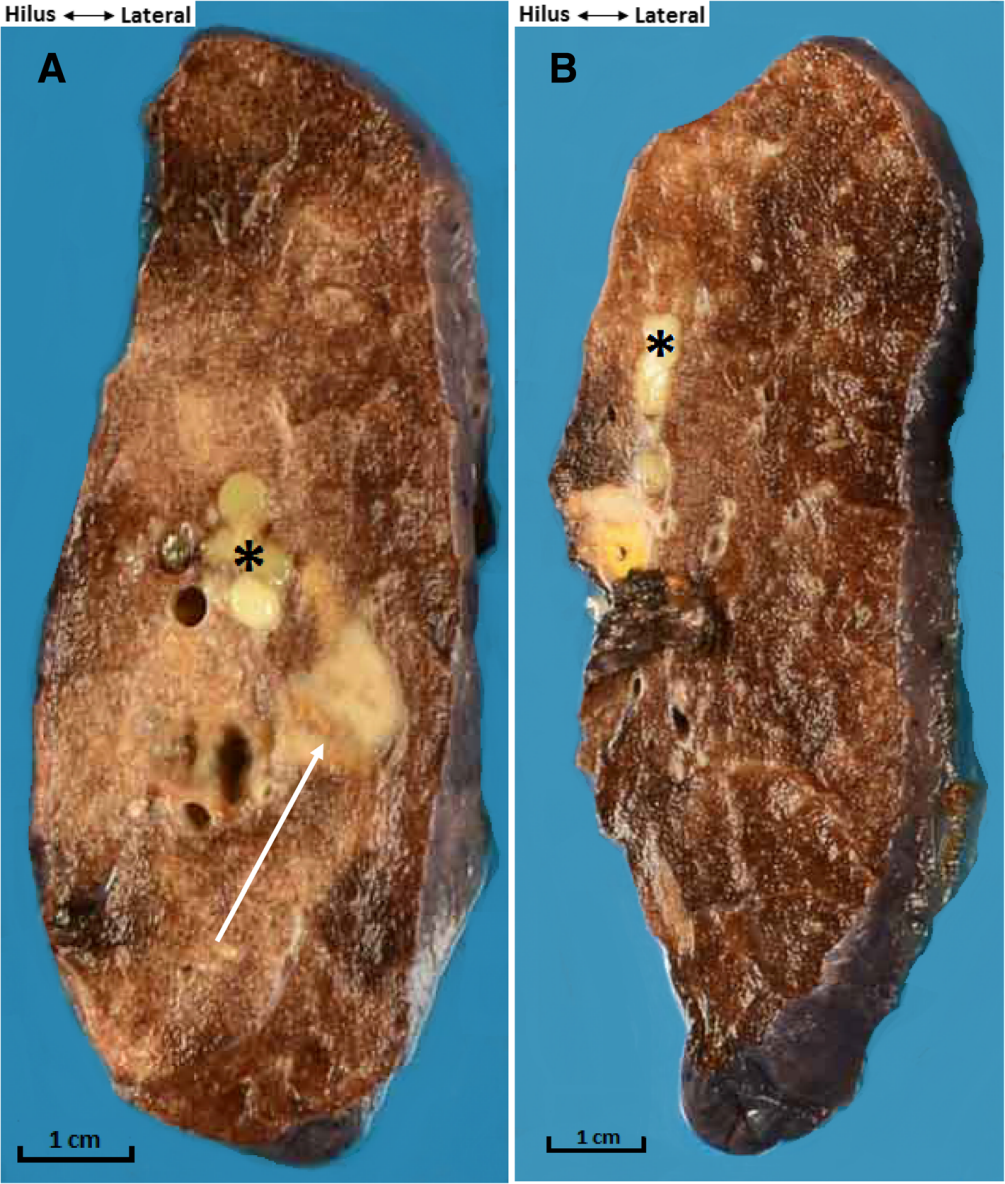
Macroscopic appearance of the resected left upper lobe shows (**a**) Perihilar atypical carcinoid (arrow) with distal bronchiectasis (asterisk). (**b**) Peripheral lung parenchyma with a subpleural consolidations (arrow) and adjacent bronchiectasis (asterisk)

**Fig. 5:**
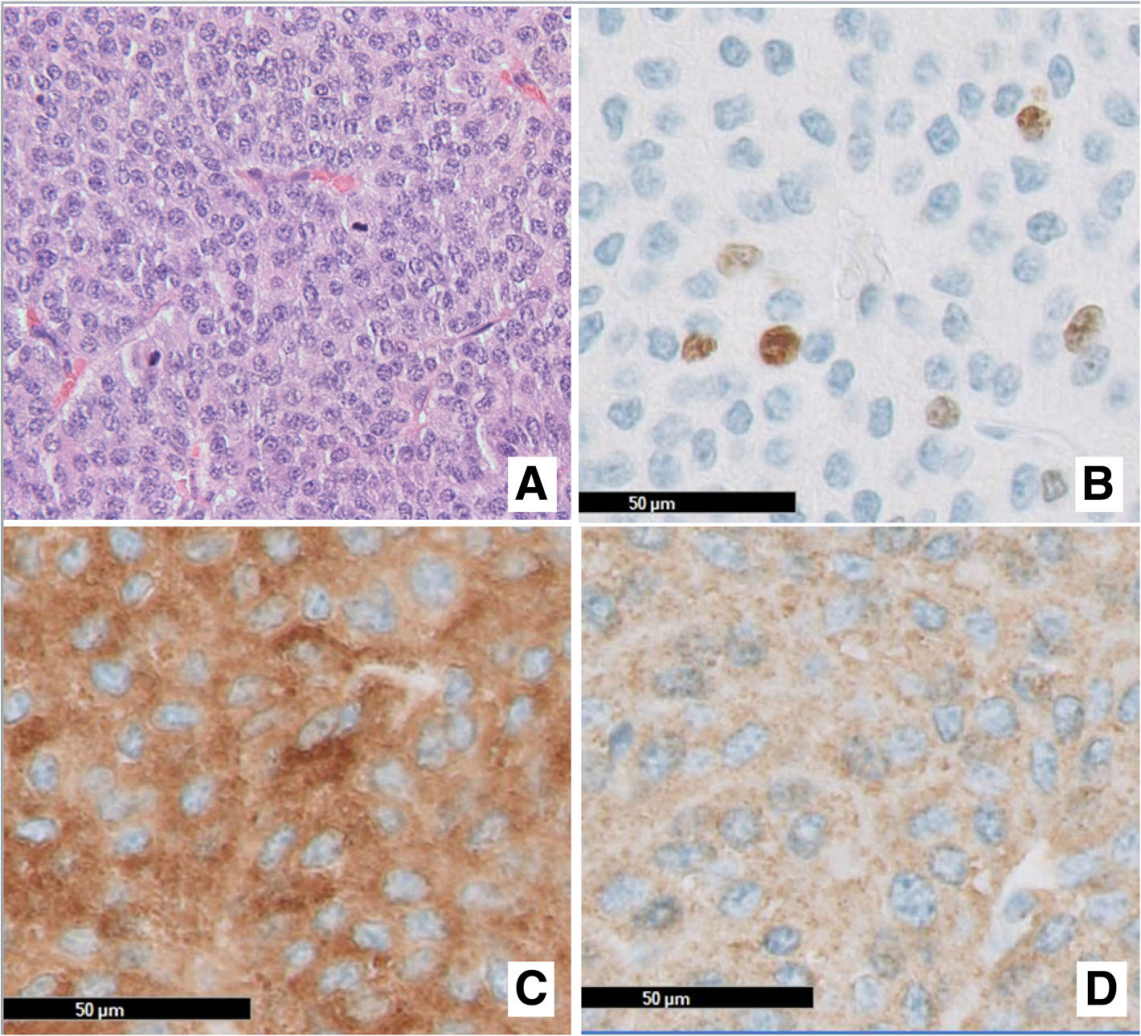
Histology of the intrabronchial tumor: (**a**) (H&E stain, × 200) at low power showing sheets of monomorphic cells with speckled chromatin. Note mitoses (5 per 2 mm2). (**a**) MIB-1, indicating a proliferation index shows about ca 5% of tumor cells in cycle. Neuroendocrine differentiation has been confirmed in synaptophysine (**c**) and chromogranin A (**d**)

**Fig. 6:**
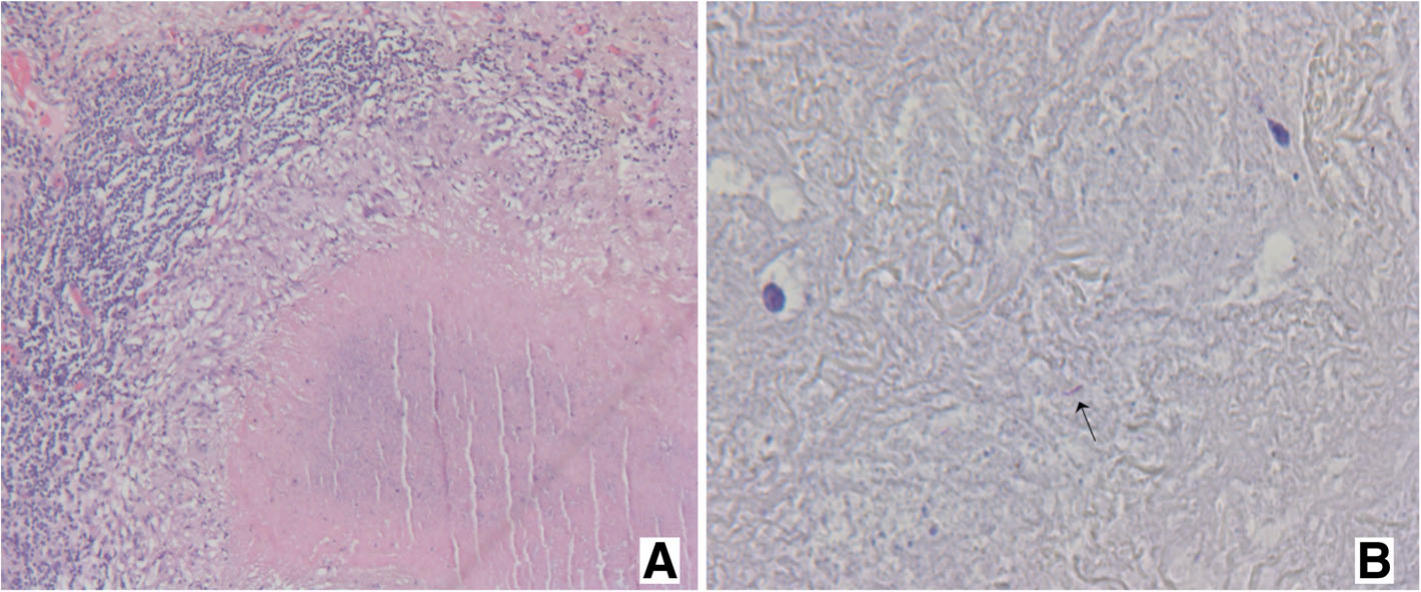
(**a**) Histology slides showing multiple consolidations distal to the carcinoid (H&E stain, × 50) and a large granuloma with central caseous necrosis, surrounded by palisading histiocytes and some giant-cells. (**b**) Ziehl-Neelsen staining × 400, demonstrating acid fast bacilli in the necrotic debris

A 45-year-old female, never smoker and without comorbidity, presented with cough, low grade fever and mild weight loss. There was no haemoptysis. She had no contact with tuberculosis patients but she had travelled to endemic countries in Asia and Africa. There was no history of recurrent infections in the past. She was diagnosed with pneumonia and treated with various courses of antibiotics but without resolution of symptoms. Physical examination revealed decreased breath sounds in the left upper lobe. Chest X-ray revealed a consolidation in the upper left hilum and left upper lobe (Fig. 1). CT scan showed a central nodular intraluminal lesion with bronchial thickening and postobstructive pneumonia in the left upper lobe. No other endobronchial lesions or focal intrapulmonary pathology was found. There was no lymphadenopathy and no pericardial or pleural effusion (Fig. 2 a, b). Bronchoscopy showed a well-defined endobronchial tumor in the apicoposterior segment of the left upper lobe. The patient was subsequently referred to our hospital for endobronchial treatment. Rigid bronchoscopy with electrocautery was attempted, but unsuccessful due to the difficult location of the lesion (Fig. 3). Subsequent left upper lobe lobectomy was uncomplicated and resulted in a radical resection, pT1bN0R0. The resected lobe showed a perihilar mass with dilation of distal bronchi that were filled with mucinous material. The peripheral lung parenchyma contained multiple ill-defined, white to yellow consolidations (Fig. 4a, b). Histology and mitotic count was consistent with atypical carcinoid (Fig. 5a, b, c, d). In the peripheral lung parenchyma, granulomatous inflammation was found (Fig. 6a). Ziehl-Neelsen staining demonstrated unequivocal acid fast bacilli (Fig. 6b). PCR for Mycobacterium genus and Mycobacterium tuberculosis (MTB) complex performed on the resection specimen were negative. Three cultures from the surgical specimen were negative for the MTB and NTM. Because tuberculosis could not be ruled out, we advised the referring hospital to start treatment with antituberculous drugs. However, at the referring hospital it was decided not to treat because of the negative PCR and culture. Since then (now 14 months of follow-up) the patient has been well, without signs of infection. CT scan of the chest during follow-up showed no signs of active tuberculosis or recurrence of the carcinoid.

## Discussion

Pulmonary carcinoids are classified according to the WHO 2015 classification as typical or atypical [4]. Typical carcinoid tumors are more common and found more centrally within the major bronchi, whereas atypical carcinoid tumors tend to arise in the periphery [5]. Bronchial carcinoid tumors are not related to smoking and they may develop more often in patients with a previous history of malignancy of the skin, urogenital tract and respiratory tract [1, 2].

Patients with typical carcinoid have a favorable prognosis with a 10-year survival of up to 80–90%, compared to 40–60% in patients with atypical carcinoids [2].

The most frequent symptoms in patients with bronchial carcinoids are cough, dyspnea and hemoptysis. Lobar obstruction often occurs in bronchial carcinoids and causes postobstructive pneumonia [6, 7]. The diagnosis is often delayed, and patients may have received several courses of antibiotics to treat recurrent pneumonia before the pulmonary carcinoid tumor is diagnosed. Endocrine manifestations occur in 1–7% of cases in addition to carcinoid syndrome [2]. The gold standard of imaging is a contrast CT scan. A chest X-ray usually reveals parenchymal changes due to obstruction [1]. Bronchoscopy is a very important tool in diagnosing bronchial carcinoid tumors. Surgical resection is the mainstay of treatment [1, 2]. In a subset of patients where the lesion is confined to the bronchial lumen, endobronchial resection can be a less invasive and parenchyma sparing alternative [8]. Systemic chemotherapy with radiotherapy can be considered in patients with advanced unresectable progressive pulmonary carcinoid [2].

The coexistence of lung cancer with tuberculosis and NTM is well known but the coexistence of bronchial carcinoid with tuberculosis or NTM is rare and has been reported previously in a few cases in the English literature.

Agave et al. reported about 9 cases of pulmonary carcinoids with MTB [9], Yilmaz et al. reported a case of a typical pulmonary carcinoid with tuberculosis in the same lobe [10] and Dixi et al. reported a case of a typical carcinoid in a lower lobe and tuberculosis in the ipsilateral upper lobe [3]. Nakamora et al. reported two cases of atypical mycobacteria with typical pulmonary carcinoids [11]. Mullick et al. reported a case of carcinoid tumor coexisting with both tubercular lesions in the same lobe, as well as regional metastasis [7]. Nagai et al. reported a case of pulmonary tumorlet with caseous granuloma associated with a NTM [12]. Kono et al. reported a case of atypical carcinoid with tuberculosis [13] (Table 1). Here, we report a case of atypical bronchial carcinoid with coexisting mycobacterial infection, either tuberculosis or NTM infection.

**Table 1:** Overview of reported cases of bronchial carcinoid and coinciding mycobacterial infection

Case Published, year	Clinical features	Radiological features
Mullick et al. (2014)	26-year-old male with low-grade fever and episodic dyspnea since one year.	Chest X-ray chest showed a few small consolidated foci in the right lower lobe. Computed tomography (CT) scan thorax showed a few nodular opacities in right lower lobe and a rounded lesion measuring 3 × 3 cm in the left lower lobe
Kono et al. (2014)	63-year-old male presented with persistent cough.	Chest X-ray demonstrated an ill-defined consolidation.
Bora et al. (2012)	28-year-old male with fever and productive cough since 10 days and 2 episodes of hemoptysis.	CT scan of the thorax revealed a well-defined round-to-oval, smoothly marginated soft tissue density located in the proximal right main stem bronchus partially obscuring its lumen. In addition, atelectasis and consolidation of right middle lobe (partial) and right lower lobe (total). Right-sided pleural effusion was also noted.
Dixit et al. (2009)	35-year-old male presented with pleuritic chest pain and low grade fever for one month.	CT scan of the thorax showed a well-defined soft-tissue density in the right lower lobe bronchus, with a few areas of calcification.
Yilmaz et al. (2004)	39-year-old female presented with chest pain for two months.	Chest radiograph showed consolidation in the right lower field. CT scan of the thorax demonstrated mediastinal lymphadenopathy and consolidation and atelectasis of the right lower lobe.
Nakamura et al. (2003)	Case 1: 81-year-old female with left lower lobe atelectasis. Case 2: 50-year-old female with atelectasis of the left upper lobe.	Case 1: Chest X ray showed left lower lobe atelectasis. Case 2: Chest X-ray and CT showed atelectasis of the left upper lobe.
Nagai et al. (1998)	73-year-old female with fever and cough and common cold symptoms.	Chest X-ray showed a consolidation in the right middle lobe.
Agaev et al. (1991)	9 cases of bronchial carcinoid and tuberculosis	

Tuberculosis has been described in association with various forms of lung malignancies. However, its association with carcinoids has been rarely reported possibly because the pulmonary carcinoid tumors are rare tumors [3].

Unfortunately, the mycobacterial culture remained negative. Several explanations are possible. First, there could be a sampling error due to uneven distribution of mycobacteria in tissue samples and/or the presence of extensive necrosis in the tissues [14]. Second, a technical problem with the culture such as the presence of a growth inhibitor could explain the negative culture. And third, the sample sent for culture could have been inadequate. In addition, there is also a remote possibility that the Ziehl-Neelsen was false positive because of contamination of the specimen by acid fast bacilli.

There are some next generation sequencing techniques emerging for the diagnosis of microorganisms in the tissue. However, a major concern is the abundance of the microorganism DNA in the tissue. Advantages of such techniques are mainly the possibility of testing multiple microorganisms in one run (multiplex testing). In our case, the low amount of the mycobacterial DNA in the necrosis likely led to false negative results using rtPCR, a lot more sensitive technique.

This case illustrates the importance of systematic examination of the resection specimen with careful attention of the lung parenchyma distal to the bronchial carcinoid. Findings such as mycobacterial infection may require additional treatment and should not be missed.

## Conclusion

Bronchial carcinoid is often associated with postobstructive bacterial pneumonia, but can sometimes also coexist with tuberculosis or NTM infection. It is therefore important not only to investigate the resected tumor, but also the obstructed lung parenchyma in the surgical resection specimen.

## Abbreviations

CT: Computed tomography
MTB: *Mycobacterium tuberculosis*
NET: Neuroendocrine tumors
NTM: Nontuberculous mycobacteria

## References

[1] Hendifar AE, Marchevsky AM, Tuli R (2017). Neuroendocrine Tumors of the Lung: Current Challenges and Advances in the Diagnosis and Management of Well-Differentiated Disease. J Thorac Oncol.

[2] Caplin ME, Baudin E, Ferolla P, Filosso P, Garcia-Yuste M, Lim E, Oberg K, Pelosi G, Perren A, Rossi RE^1^,Travis WD; ENETS consensus conference participants. Pulmonary neuroendocrine (carcinoid) tumors: European neuroendocrine tumor society expert consensus and recommendations for best practice for typical and atypical pulmonary carcinoids. Ann Oncol 2015. 26(8):1604–1620. 10.1093/annonc/mdv041. Epub 2015 Feb 2. 10.1093/annonc/mdv041 25646366

[3] Dixit R, Gupta R, Yadav A, Paramez AR, Sen G (2009). Sharma S. A case of pulmonary carcinoid tumor with concomitant tuberculosis. Lung India : Official Organ of Indian Chest Society.

[4] Pelosi G, Sonzogni A, Harari S (2017). Classification of pulmonary neuroendocrine tumors: new insights. Translational Lung Cancer Research.

[5] Waheed Z, Irfan M, Fatimi S, Shahid R (2013). Bronchial carcinoid presenting *as* multiple lung abscesses. Journal of the College of Physicians and Surgeons Pakistan.

[6] Bora MK, Vithiavathi S (2012). Primary bronchial carcinoid: a rare differential diagnosis of pulmonary Koch in young adult patient. Lung India : Official Organ of Indian Chest Society..

[7] Mullick S, Gupta K, Dewan R, Gupta K. Typical bronchial carcinoid with local metastasis and coexisting tuberculosis in the same lung: a case report. The Journal of Association of Chest Physicians. 2014. 10.4103/2320-8775.135120.

[8] Reuling EMBP, Dickhoff C, Plaisier PW, Coupé VMH, Mazairac AHA, Lely RJ, Bonjer HJ, Daniels JMA (2018). Endobronchial treatment for bronchial carcinoid: patient selection and predictors of outcome. Respiration.

[9] Agaev FF (1991). The diagnosis and treatment of bronchial carcinoids. Grud Serdechnososudistaia Khir.

[10] Yilmaz A, Güngör S, Damadolu E, Aksoy F, Aybatli A, Düzgün S (2004). Coexisting bronchial carcinoid tumor and pulmonary tuberculosis in the same lobe: a case report. Tuberk Toraks.

[11] Nakamura Y, Okada Y, Endo C, Aikawa H, Sakurada A, Sato M, Kondo T (2003). Endobronchial carcinoid tumor combined with pulmonary non-tuberculous mycobacterial infection: report of two cases. Lung Cancer.

[12] Nagai S, Katakura H, Okazaki T, Ishida H, Wazawa H, Hanawa T (1998). A pulmonary tumorlet with caseous granuloma associated with atypical mycobacterium. Nihon Kokyuki Gakkai Zasshi.

[13] Kono et al (2014). A case of pulmonary carcinoid tumor with tuberculosis. AJRS.

[14] Chawla K, Gupta S, Mukhopadhyay C, Rao PS, Bhat SS. PCR for M. tuberculosis in tissue samples. J Infect Dev Ctries 2009 Mar 1;3(2):83–87. doi: 10.3855/jidc.53 PMID:19755735. 10.3855/jidc.53 19755735

