# Correction to: Atypical bronchial carcinoid with postobstructive mycobacterial infection: case report and review of literature

**DOI:** 10.1186/s12890-019-0988-2

**Published:** 2019-11-26

**Authors:** Abdulrahman Hakami, Evita Zwartkruis, Teodora Radonic, Johannes M. A. Daniels

**Affiliations:** 1 Department of Pulmonary Medicine, Amsterdam University Medical Center, Amsterdam, The Netherlands; 2 Department of Medicine, College of Medicine, Jazan University, Jazan, Saudi Arabia; 3 Department of Pathology, Amsterdam University Medical Center, Amsterdam, The Netherlands

**Correction to: BMC Pulm Med**

**https://doi.org/10.1186/s12890-019-0806-x**

Please note that an affiliation has been missed from the published article [1].

The missing affiliation is:

2 - Department of Medicine, College of Medicine, Jazan University, Jazan, Saudi Arabia.

The corresponding author should have this affiliation.

Please find the corrected author list and list of affiliations in this article.

The authors apologize for any inconvenience caused.
